# Patent Medicines

**Published:** 1874-04

**Authors:** M. S. Bidwell


### PATENT MEDICINES.

M. S. BIDWELL.

The question is often asked, "Why are these humbugs so universally used?" We may answer, in general terms, that they are used because the mass of mankind do not treat themselves intelligently. Three centuries ago it was generally believed that the touch of a king's hand could cure scrofula, and that the cure would be permanent only while the coin given by the same hand was suspended from the patient's neck. That delusion may have become obsolete, but the same spirit remains in full force at the present day. Intelligent men look upon medicines only as one of the means by which the skillful physician seeks to remedy the diseased condition of the body; but to the world in general the doctor stands pretty much on the same footing as the African Fetish-man, and if his charms fail to cast out the evil spirit, he is denounced as a false prophet, and the sufferer seeks some other spell or incantation by which to get relief. "His medicines didn't cure me," is considered a complete condemnation, when, perhaps, the medicine may have been entirely subordinate in the physician's plan of treatment, and his instructions in matters of vital importance may have been wholly neglected. This error of considering medicines as the real and only means of cure, and supposing them to act as a charm or spell in exorcising disease, appears to lie at the basis of the quack's success. The Patent Medicine proprietor, skillfully availing himself of this delusion, sets forth his remedies as possessing just this magic efficacy. Like the sword of Solomon in the Arabian Nights, bearing the magic word "power," before which the Genii of earth and air were subdued, so at the touch of Dr. Fraud's Magic Cure, (if you will take his word for it,) the demons of disease will fall helpless or flee in confusion—and the public accept his claim.

Of course, too, if the patient thinks nothing of the physician's skill and care, but rests his faith entirely upon the mysterious powers of the medicine, he will naturally look upon the doctor himself as a useless expense. Is it not a fact that most people, if relieved of a serious illness by skillful treatment without the use of medicines, will consider the doctor's bill an imposition? To put it in another shape, they estimate the value of his services by the amount of medicines he gives, and if relieved without drugs, they feel as if they had not got the worth of their money. Hence the Patent Medicines appeal to the desire of the unreflecting public to be cured cheaply.

Here again the delusion is often fostered not only by the maker of these articles, but by the retailer. The merchant is tempted to recommend them, partly because he often does not know any better, and partly from the instinctive mercantile desire to sell his goods. As regards the druggists, who are the largest, though by no means the only venders of these articles, it is gratifying to know that this is becoming less and less true, every year, as professional honor and professional knowledge are increasing. The writer can say with much satisfaction, that during an experience of several years in the drug business, he has never recommended or advised the use of any secret remedy; and all the leading Pharmacists in this country stands upon the same grounds.

If there is any one class that are more responsible than any other for this abuse of popular ignorance, it is the doctors—those same regular practitioners, who so earnestly denounce the quacks and warn the public against them. This may seem a startling assertion, but its truth will appear from the following considerations. In the first place, almost all Patent Medicines are originally put forth, and a majority of them are now owned and run by physicians. The mere mention of the names of Drs. Jayne, Herrick, Pierce and Walker, will illustrate this fact; and a careful examination of the proprietary articles in any drug store will show conclusively that a majority of them are controlled by M. D.'s, who do not fail to claim superior excellence for their wares on account of their professional standing. In the next place, they are all, without any exception, endorsed and recommended by physicians. Open any Patent Medecine Almanac and you will find certificates of this character *usque ad nauseam.* Some of these may be fictitious, but they need not be, for (in the third place,) almost every physician does recommend some one or more of these nostrums, although he may denounce them as a class. Since then these medicines are mostly put forth by the doctors, are all recommended by doctors, and are sold mainly on their recommendation, is it not fair to say that the physicians are largely responsible for the extent and success of the Patent Medicine trade ?

The further questions—" Why should Patent Medicines not be used ?"—and—" How may their use be discouraged ?" may perhaps be discussed on another occasion.

